# Influences of ethanol on the structure of toxic trans-crotonaldehyde in mitochondria coming from rat myocardium

**DOI:** 10.1038/s41598-017-09656-6

**Published:** 2017-08-30

**Authors:** Yanbin Su, Xiaowei Ma, Yanwen Su, Tongxing Mu, Yanhe Li, Ning Jiang, Yuyun Su, Qi Zhang

**Affiliations:** 1 College of Chemical & Pharmaceutical Engineering, Jilin University of Chemical Technology, Jilin, 132022 China; 2 Department of Civil Engineering, Tsinghua University, Beijing, 100084 China; 3 Lunan Pharmaceutical Group Corporation, Linyi, 276005 China

## Abstract

Inappropriate use of ethanol (EtOH) had led to noticeable health problems, but a beneficial phenomenon was found that EtOH displayed unique influences for toxic trans-crotonaldehyde (TCA) derived from mitochondrial lipid peroxidation. The influences of EtOH on the structure of TCA were systematically probed by UV-vis & Raman spectroscopy in the absence and presence of mitochondria, respectively. The maximum UV-vis peak at 301 nm of TCA was red shifted by hydroxyl (-OH) and methyl (-CH_3_) of EtOH, respectively. Raman stretching band of aldehyde (-CH=O) of TCA (TCA-CH=O) was split by the -CH_3_ of EtOH. The -CH_3_ increased TCA-CH=O stretching frequency while the -OH induced it. The more exposed -OH, the less stretching frequency. The ectopic -CH_3_ red shifted the UV-vis peak at 301 nm and Raman band of TCA-CH=O. In mitochondria, EtOH red shifted Raman stretching band of TCA-CH=O. Raman stretching bands of C-H, C-O and C-C of EtOH were red shifted, while Raman stretching bands of -CH_2_ and C-C-O of EtOH disappeared. The paper unearths the influences of EtOH to trap and transform the structure of TCA-CH=O. This discovery has an important contribution to eliminate TCA in order to protect and repair mtDNA by means of the decrease of 8-oxoG.

## Introduction

Although inappropriate use of ethanol (EtOH) had led to noticeable health problems, our research team accidentally discovered a beneficial phenomenon that EtOH showed a unique influence on toxic trans-crotonaldehyde (TCA) derived from mitochondrial lipid peroxidation. However, the detailed molecular mechanism and potential significance of EtOH on TCA is unknown so far.

**Figure 1:**
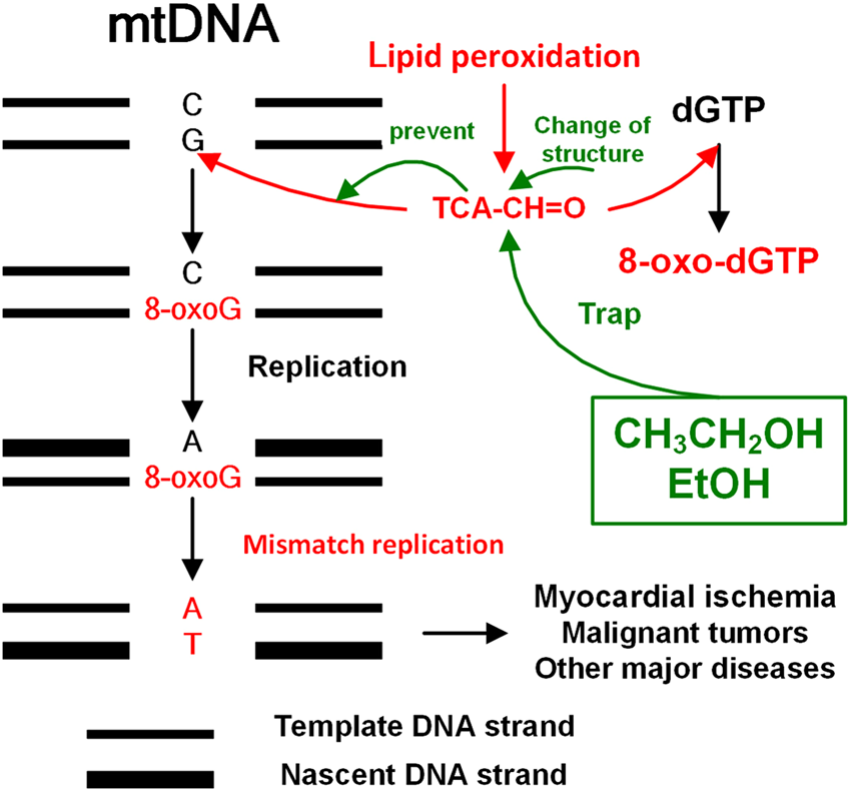
The mode of action of EtOH-induced prevention of mismatch replication.

TCA is a potentially and dangerously mutagenic compound to mitochondrial DNA (mtDNA) *in vitro* and *in vivo* ^1, 2^. In all nucleobases, aldehyde (-CH=O) of TCA (TCA-CH=O) possibly attacks various nucleic acids, but guanine (G) is the most susceptible to TCA-CH=O^3^. When TCA is close to mtDNA^4, 5^, the G is converted to 8-oxoguanine (8-oxoG) which can pair with adenine (A) as well as cytosine (C), then further converts into thymine (T), A, or C^6, 7^. The 8-oxoG is the major oxidized base in mtDNA^8, 9^. If the 8-oxoG is persistently generated, the accumulation of 8-oxoG will accelerate the transformation of G to T in mtDNA. Under this circumstance, it is inevitable that the development and progression of some major diseases such as myocardial ischemia and malignant tumors (Fig. 1)^10–12^. Therefore, the elimination of TCA can be effective in preventing the formation and accumulation of the 8-oxoG in mtDNA. However, the elimination method of TCA has not been reported at present.

The primary sources of TCA include lipid peroxidation in mitochondria *in vivo* ^13, 14^, tobacco tar^15^, and environmental pollution *in vitro*, etc^16^. In search of how to eliminate the mutagenic action of TCA, it was accidentally discovered that the maximum UV-vis peak at 301 nm of TCA was red-shifted by EtOH. Research finding prompted the idea that EtOH has been probably involved in the prevention and repair of mtDNA damage via the structural change of TCA-CH=O^17^. The influences of EtOH on the molecular structure of TCA-CH=O provide useful information on how to eliminate TCA. The purpose of this study was to explore the influences of EtOH on the structure of TCA-CH=O, especially in mitochondria.

EtOH includes hydroxyl (-OH) and methyl (-CH_3_) while TCA contains -CH=O and carbon-carbon double bond (C=C). According to the characteristics of Raman spectrum detection, Raman band accurately reflects the oscillatory changes of a particular functional group in their microenvironment. Raman vibrational motions are divided into self-rotation motion and translational motion of a particular functional group. The latter is generally far shorter than the former. Raman spectra are mainly derived from translational motion. In order to clarify the molecular behaviors of EtOH interacted with TCA-CH=O, research designs were carried out as follows. Firstly, UV-vis spectroscopic behaviors of EtOH, -OH, and -CH_3_ on TCA-CH=O were observed; Secondly, Raman spectroscopic behaviors of EtOH, -OH, and -CH_3_ on the structure of TCA-CH=O were probed; Finally, in mitochondria, Raman spectroscopic changes of TCA and EtOH themselves during the interaction of EtOH with TCA-CH=O were studied.

## Results and Discussion

### UV-vis spectroscopic behaviors of EtOH, -OH, and -CH_3_ on TCA

**Figure 2:**
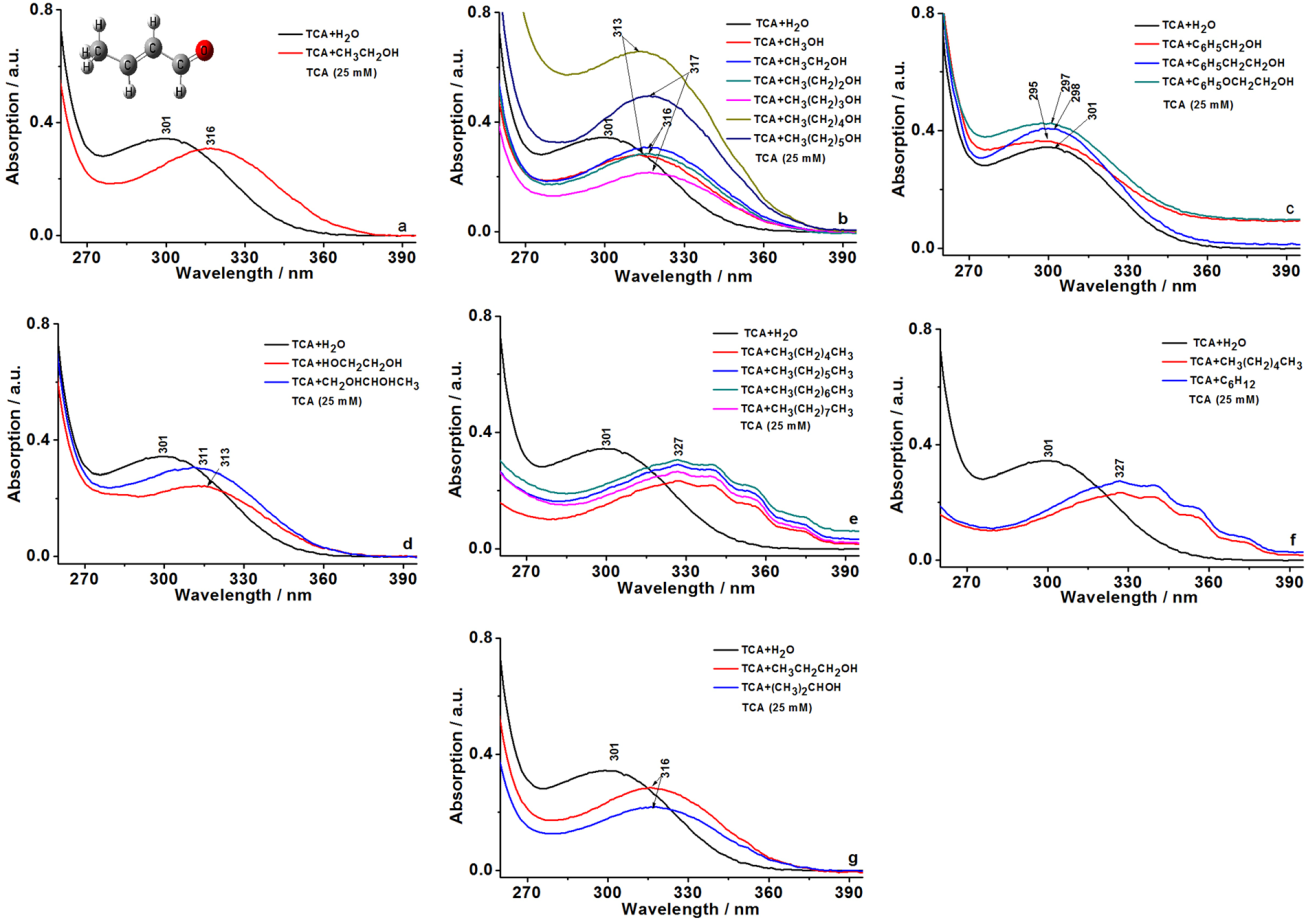
UV-vis spectroscopic behaviors of EtOH, -OH, and -CH_3_ on TCA. (**a)** The maximum UV-vis peaks of TCA was located 301 nm (solvent: ultra pure water, H_2_O) and 316 nm (solvent: EtOH, CH_3_CH_2_OH, 99.7%), respectively. (**b**) UV-vis spectroscopic behaviors of ultra pure water (H_2_O), methanol (CH_3_OH, 99%), EtOH (CH_3_CH_2_OH, 99.7%), n-propyl alcohol (CH_3_(CH_2_)_2_OH, 99%), n-butyl alcohol(CH_3_(CH_2_)_3_OH, 99%), n-pentanol (CH_3_(CH_2_)_4_OH, 99%), and n-hexyl alcohol (CH_3_(CH_2_)_5_OH, 99%) on TCA (97%), respectively. (**c**) UV-vis spectroscopic behaviors of ultra pure water (H_2_O), benzyl alcohol (C_6_H_5_CH_2_OH, 99%), 2-phenylethanol (C_6_H_5_CH_2_CH_2_OH, 99%), phenoxyethanol (C_6_H_5_OCH_2_CH_2_OH, 99%) on TCA (97%), respectively. (**d**) UV-vis spectroscopic behaviors of ultra pure water (H_2_O), glycol (HOCH_2_CH_2_OH, 99%), and glycerol (HOCH_2_CHOHCH_2_OH, 99%) on TCA (97%), respectively. (**e**) UV-vis spectroscopic behaviors of ultra pure water (H_2_O), n-hexane (CH_3_(CH_2_)_4_CH_3_, 99%), n-heptane (CH_3_(CH_2_)_5_CH_3_, 99%), n-octane (CH_3_(CH_2_)_6_CH_3_, 99%), and n-nonane (CH_3_(CH_2_)_7_CH_3_, 99%) on TCA (97%), respectively. (**f**) UV-vis spectroscopic behaviors of ultra pure water (H_2_O), n-hexane (CH_3_(CH_2_)_4_CH_3_, 99%), and cyclohexane (C_6_H_12_, 99%) on TCA (97%), respectively. (**g**) UV-vis spectroscopic behaviors of ultra pure water (H_2_O), n-propyl alcohol (CH_3_CH_2_CH_2_OH, 99%), and isopropanol ((CH_3_)_2_CHOH, 99%) on TCA (97%), respectively.

Using ultrapure water as the control solvent, the maximum UV-vis peak at 301 nm of TCA as the reference, UV-vis spectroscopic behaviors of EtOH, -OH, and -CH_3_ on TCA display in Fig. 2a–g.

Figure 2a,b and d show that EtOH like the other aliphatic alcohols and polylols significantly red shifted UV-vis peak at 301 nm of TCA. But the aromatic alcohols blue shifted UV-vis peak at 301 nm of TCA (Fig. 2c). Observations show that TCA interacts differently with aromatic groups than with -CH_3_. Figure 2e,f and g illustrate that alkanes including cyclic methylation and ectopic -CH_3_ obviously red shifted UV-vis peak at 301 nm of TCA. If the -CH_3_ was replaced by different aromatic groups, the spectroscopic behaviors of aromatic alcohols on TCA displayed blue shift of UV-vis peak at 301 nm of TCA. These findings provide the important molecular trails for finding methods to trap or transform the structure of TCA. In order to further elucidate the spectroscopic behaviors of both -CH_3_ and -OH of EtOH on TCA, the spectroscopic behaviors of both -CH_3_ and -OH of EtOH on TCA were explored by means of Raman spectroscopy.

The toxic targets of TCA are both -CH=O and C=C functional groups which are responsible for its negative influence. The Raman bands of -CH=O and C=C are situated at 1688 cm^−1^ and 1641 cm^−1^, respectively. The Raman band changes of TCA-CH=O in different microenvironments depend not only on the H delocalization of TCA-CH=O itself, but also on the active -OH of exogenous Et-OH^18^. Therefore, the position, shape, and full width at half-maximum (FWHM) changes of Raman bands of TCA-CH=O could clearly describe the influences of EtOH on the structure of TCA-CH=O.

### Gross Raman spectroscopic behaviors of EtOH on TCA-CH=O in the absence and presence of mitochondria

**Figure 3:**
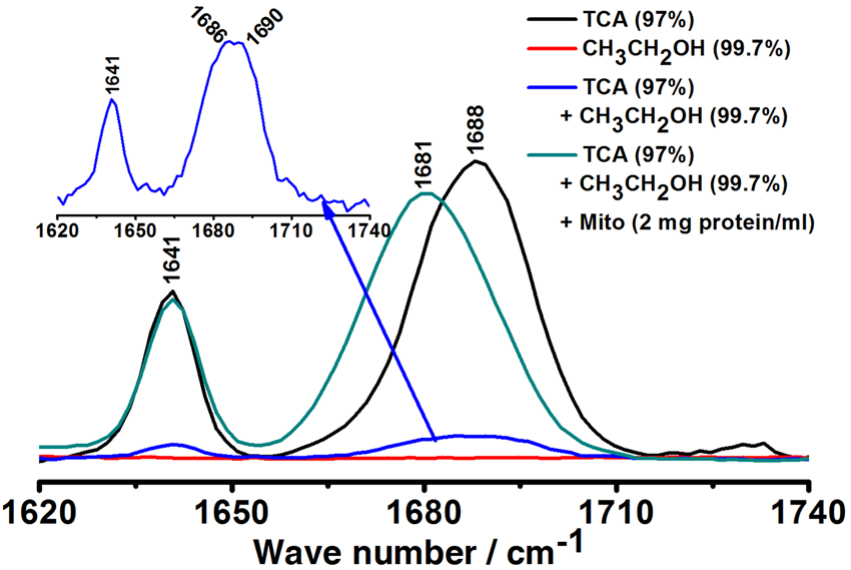
Gross Raman spectroscopic behaviors of EtOH on TCA-CH=O in the absence and presence of mitochondria. The shapes of Raman bands at 1688 cm^−1^ and 1641 cm^−1^ of TCA are symmetric without split (the black line in Fig. 3). Compared with Raman spectra of TCA and EtOH (the red line in Fig. 3), respectively, in the absence of mitochondria, the Raman band of 1688 cm^−1^ assigned to -CH=O was split (see also Fig. 4b) by EtOH with significantly decreased intensity (the blue line in Fig. 3). In the presence of mitochondria, the Raman band of 1688 cm^−1^ assigned to -CH=O was remarkably shifted from 1688 cm^−1^ to 1681 cm^−1^ by EtOH (the green line in Fig. 3). The above results show that EtOH has an important influences on the molecular structure of TCA-CH=O, especially in mitochondria.

**Figure 4:**
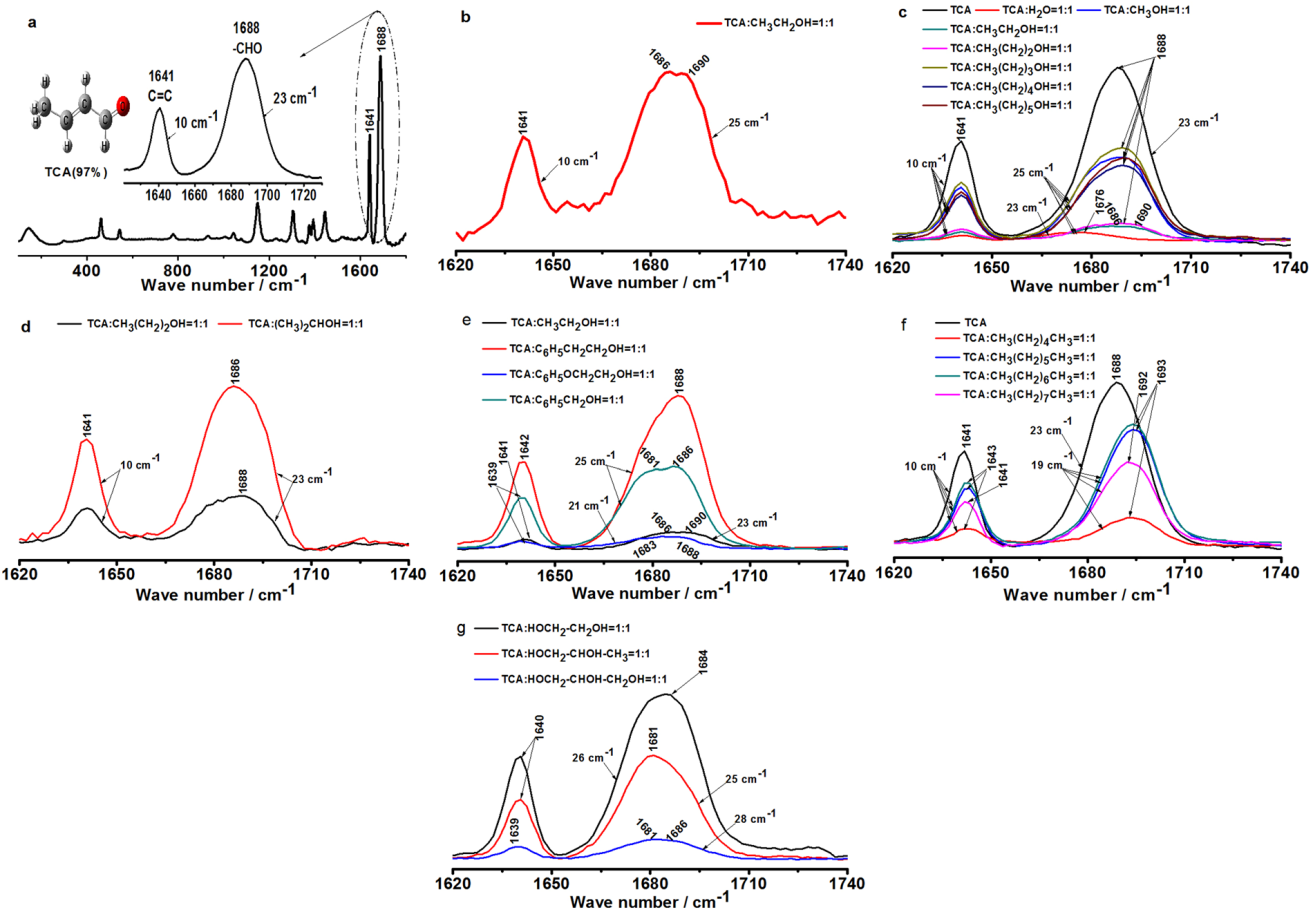
Raman spectroscopic behaviors of EtOH, -OH, and -CH_3_ on TCA. (**a**) The characteristic Raman bands of TCA were located at 1688 cm^−1^ assigned to -CH=O and 1641 cm^−1^ assigned to C=C, respectively. The standard FWHMs of above mentioned Raman bands were 23 cm^−1^ and 10 cm^−1^, respectively. (**b**) Raman spectroscopic behaviors of EtOH (CH_3_CH_2_OH, 99.7%) on TCA-CH=O (97%) in the position, shape, and FWHMs of bands at 1688 cm^−1^ and 1641 cm^−1^, respectively. (**c**) Raman spectroscopic behaviors of methanol (CH_3_OH, 99%), EtOH (CH_3_CH_2_OH, 99.7%), n-propyl alcohol (CH_3_(CH_2_)_2_OH, 99%), n-butyl alcohol(CH_3_(CH_2_)_3_OH, 99%), n-pentanol(CH_3_(CH_2_)_4_OH, 99%), and n-hexyl alcohol (CH_3_(CH_2_)_5_OH, 99%) on TCA-CH=O (97%) in the position, shape, and FWHM of bands at 1688 cm^−1^ and 1641 cm^−1^, respectively. (**d**) Raman spectroscopic behaviors of n-propyl alcohol (CH_3_(CH_2_)_2_OH, 99%) and isopropanol (CH_3_(CH_2_)_2_OH, 99%), on TCA-CH=O (97%) in the position, shape, and FWHM of bands at 1688 cm^−1^ and 1641 cm^−1^, respectively. (**e**) Raman spectroscopic behaviors of EtOH (CH_3_CH_2_OH, 99.7%), 2-phenylethanol (C_6_H_5_CH_2_CH_2_OH, 99%), phenoxyethanol (C_6_H_5_OCH_2_CH_2_OH, 99%), and benzyl alcohol (C_6_H_5_CH_2_OH, 99%) on TCA-CH=O (97%) in the position, shape, and FWHM of bands at 1688 cm^−1^ and 1641 cm^−1^, respectively. (**f**) Raman spectroscopic behaviors of n-hexane (CH_3_(CH_2_)_4_CH_3_, 99%), n-heptane (CH_3_(CH_2_)_5_CH_3_, 99%), n-octane (CH_3_(CH_2_)_6_CH_3_, 99%), and n-nonane (CH_3_(CH_2_)_7_CH_3_, 99%) on TCA-CH=O (97%) in the position, shape, and FWHM of bands at 1688 cm^−1^ and 1641 cm^−1^, respectively. (**g**) Raman spectroscopic behaviors of glycol (HOCH_2_CH_2_OH, 99%), 1, 2- propylene glycol (HOCH_2_CHOHCH_3_, 99%), and glycerol (HOCH_2_CHOHCH_2_OH, 99%) on TCA-CH=O (97%) in the position, shape, and FWHM of bands at 1688 cm^−1^ and 1641 cm^−1^, respectively.

Raman spectra of TCA and TCA-CH=O^19^ are shown in Fig. 3 and Figure S1 in supporting information. In the range of 100 cm^−1^ to 1800 cm^−1^, there are altogether 15 Raman bands in TCA. Among them, the 1688 cm^−1^ and 1641 cm^−1^ represent the -CH=O and C=C stretching mode of TCA^20^, respectively. The shapes of Raman bands at 1688 cm^−1^ and 1641 cm^−1^ of TCA are symmetric without split. However, Fig. 3 shows that compared with Raman spectra of TCA (the black line in Fig. 3) and EtOH (the red line in Fig. 3), respectively, in the absence of mitochondria, the Raman band of 1688 cm^−1^ assigned to -CH=O was split (see also Fig. 4b) by EtOH with significantly decreased intensity (the blue line in Fig. 3). But in the presence of mitochondria, the Raman band of 1688 cm^−1^ assigned to -CH=O was remarkably shifted from 1688 cm^−1^ to 1681 cm^−1^ by EtOH (the green line in Fig. 3). The above results show that EtOH has an important influences on the molecular structure of TCA-CH=O, especially in mitochondria.

### Raman spectroscopic behaviors of EtOH, -OH, and -CH_3_ on the structure of TCA-CH=O

Raman spectroscopic behaviors of EtOH interacting with TCA-CH=O can be clearly described according to the characteristic changes of position, shape, and FWHM of corresponding Raman bands. The characteristic Raman bands of TCA were located at 1688 cm^−1^ assigned to -CH=O and 1641 cm^−1^ assigned to C=C, respectively. The normal FWHMs of Raman bands of both TCA-CH=O and TCA-C=C were 23 cm^−1^ and 10 cm^−1^ (Fig. 4a), respectively. Taking Raman band at 1688 cm^−1^ of TCA as the observed band, EtOH not only led to the split of band from 1686 cm^−1^ to 1690 cm^−1^ but also broadened the FWHM of band from 23 cm^−1^ to 25 cm^−1^ (Fig. 4b). The influences of other aliphatic alcohols on TCA displayed only the increase of FWHM of band without split of band (Fig. 4c). The isopropanol slightly red shifted the band without the change of FWHM of band(Fig. 4d). Raman spectroscopic behaviors of ectopic -CH_3_ of aliphatic alcohols on TCA differed from the -CH_3_ of aliphatic alcohols. Compared with EtOH (the black line in Fig. 4e), 2-phenylethanol eliminated the split of band (the red line in Fig. 4e), while phenoxyethanol (the blue line in Fig. 4e) and benzyl alcohol (the green line in Fig. 4e) further red shifted band with split. By careful analysis of this phenomenon, it was founded that the split of band at 1688 cm^−1^ of TCA by EtOH was closely related to the -CH_3_ or H in -CH_3_. When the phenyl substituted H in -CH_3_, the split of band disappear. However, when the phenoxy substituted H in -CH_3_ or the phenyl substituted -CH_3_, the split of band slightly expanded with red shift. Moreover, the FWHM of band was broadened by 2-phenylethanol and benzyl alcohol, while the FWHM of band was narrowed by phenoxyethanol. The results show that FWHM of band was also closely related to the -CH_3_ or H in -CH_3_. The Raman band at 1688 cm^−1^ of TCA was significantly blue shifted by alkanes. The displacement are 4 cm^−1^ to 5 cm^−1^ (Fig. 4f). The FWHM of band was simultaneously decreased 4 cm^−1^ from 23 cm^−1^ to 19 cm^−1^. Both blue shift and decrease of FWHM of band become the spectroscopic behaviors of -CH_3_ hydrophobic and steric on the structure of TCA-CH=O. Results show that the C-H group can interact with the molecular environment via van-der-Waals force. The glycol and 1, 2-propylene glycol enhanced the red shift of band from 1688 cm^−1^ to 1684 cm^−1^ and 1681 cm^−1^, respectively. The glycerol not only strengthened the red shift of band from 1688 cm^−1^ to 1681 cm^−1^, but also broadened the split of band. Besides, the FWHM of band was widened by the polylols, from 23 cm^−1^ to 26 cm^−1^, 25 cm^−1^, and 28 cm^−1^, respectively (Fig. 4g). The analyses recommend that two adjacent -OH enhanced only red shift of band at 1688 cm^−1^ while three adjacent -OH not only promoted the red shift of band at 1688 cm^−1^ but also broadened the split of band at 1688 cm^−1^. The more exposed -OH, the wider FWHM or the less stretching frequency of band at 1688 cm^−1^.

Brief summary, Raman spectral behaviors of both -OH and -CH_3_ of EtOH on TCA-CH=O can be described as follows. EtOH brings about the split of Raman band of TCA-CH=O at 1688 cm^−1^ related to the -CH_3_ or H in -CH_3_. The -CH_3_ blue shifts the Raman band of TCA-CH=O, while the -OH red shifts it. The -CH_3_ reduces the FWHM of Raman band, while the -OH broadens it. The more exposed active -OH in polyhydroxy alcohols, the wider FWHM or the less stretching frequency of Raman bands of TCA-CH=O. The ectopic -CH_3_ only red shifts the Raman band of TCA-CH=O. The spectroscopic behavior of -CH_3_ of EtOH on TCA-CH=O is obviously opposite to that of -OH of EtOH.

### Raman spectroscopic changes of TCA and EtOH during the interaction of EtOH with TCA in mitochondria

**Figure 5:**
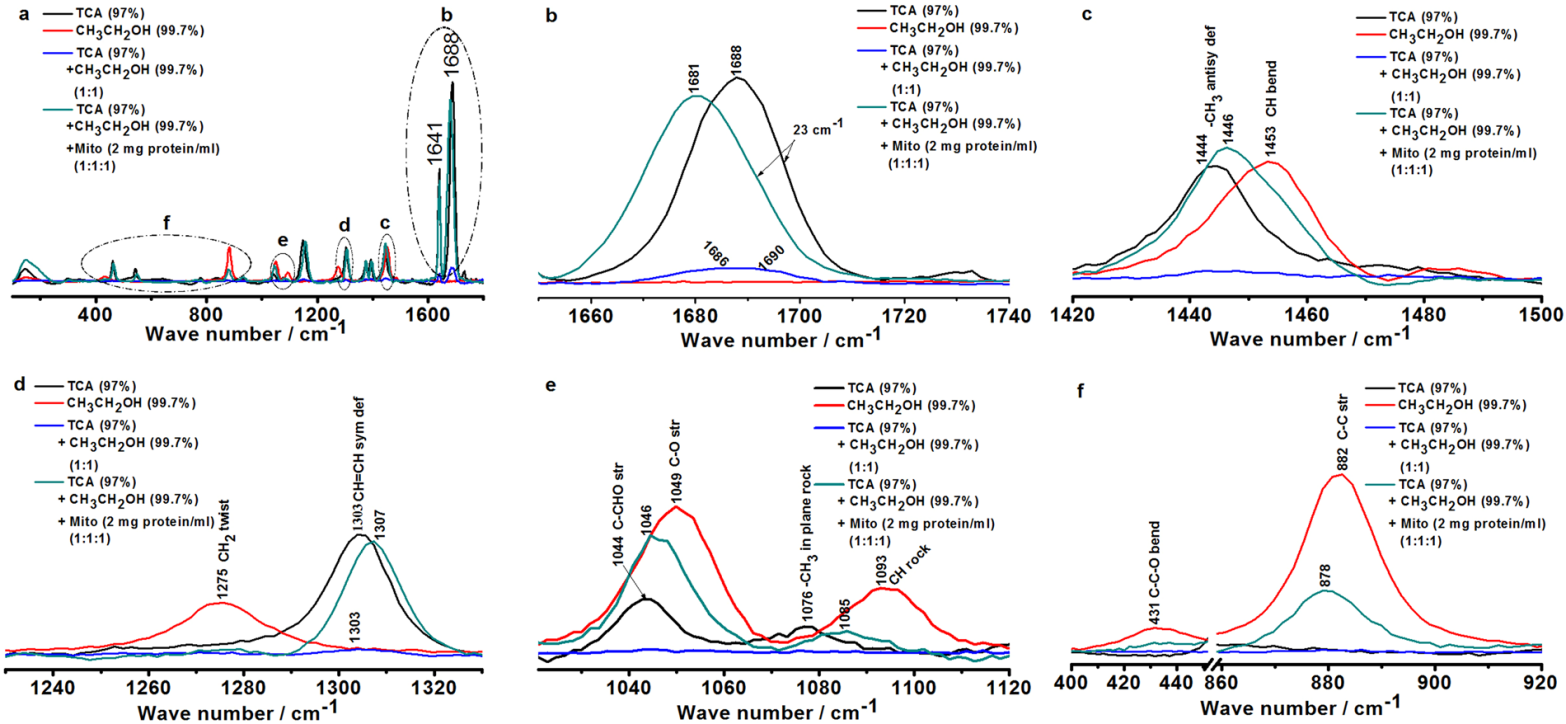
Raman spectroscopic changes of TCA and EtOH during the interaction of EtOH with TCA in mitochondria. (**a**) Gross Raman spectroscopic changes of TCA and EtOH in the range of 100 cm^−1^ to 1800 cm^−1^ during the interaction of EtOH with TCA in mitochondria. (**b**) Characteristic Raman spectroscopic changes of TCA and EtOH in the range of 1650 cm^−1^ to 1740 cm^−1^ during the interaction of EtOH with TCA in mitochondria. (**c**) Characteristic Raman spectroscopic changes of TCA and EtOH in the range of 1420 cm^−1^ to 1500 cm^−1^ during the interaction of EtOH with TCA in mitochondria. (**d**) Characteristic Raman spectroscopic changes of TCA and EtOH in the range of 1230 cm^−1^ to 1330 cm^−1^ during the interaction of EtOH with TCA in mitochondria. (**e**) Characteristic Raman spectroscopic changes of TCA and EtOH in the range of 1020 cm^−1^ to 1120 cm^−1^ during the interaction of EtOH with TCA in mitochondria. (**f)** Characteristic Raman spectroscopic changes of TCA and EtOH in the range of 400 cm^−1^ to 920 cm^−1^ during the interaction of EtOH with TCA in mitochondria.

Raman spectrum of TCA displays the black line in Fig. 5. Raman spectrum of EtOH shows six specific bands in the investigated frequency range (the red line in Fig. 5)^21, 22^. The 1453 cm^−1^, 1275 cm^−1^, and 1093 cm^−1^ correspond to the CH bend, CH_2_ twist, and CH rock mode, respectively. The 1049 cm^−1^, 882 cm^−1^, and 431 cm^−1^ represent the C-O stretching, C-C stretching, and C-C-O bend^23–25^, respectively.

Raman spectroscopic behaviors of EtOH on TCA illustrates the in blue line Fig. 5. In mitochondria, Raman spectroscopic behaviors of EtOH on TCA only red shifted the band at 1688 cm^−1^, from 1688 cm^−1^ to 1681 cm^−1^ (the green line in Fig. 5). From the comprehensive analysis of the above Raman spectra, the molecular target of EtOH on TCA is no doubt the -CH=O of TCA. Therefore, Raman spectroscopic behaviors of TCA during the interaction of EtOH with TCA are clear and definite for the first time.

From the green line in Fig. 5, Raman spectroscopic behaviors of EtOH during the interaction of EtOH with TCA in mitochondria are as follows. The 1453 cm^−1^, 1093 cm^−1^, 1049 cm^−1^, and 882 cm^−1^ were red shifted, while the 1275 cm^−1^ and 431 cm^−1^ disappeared. Raman spectroscopic behaviors of EtOH itself were the red shift of C-H bend and rock mode, C-O stretching, C-C stretching, and the disappearance of CH_2_ twist and C-C-O bend. At the same time, Raman spectroscopic behaviors of TCA as toxic target were red shift of the band at 1688 cm^−1^ assigned to -CH=O with blue shift of the bands at 1444 cm^−1^ assigned to -CH_3_, 1303 cm^−1^ assigned to CH=CH, and 1044 cm^−1^ assigned to C-CH=O, respectively.

Generally speaking, the red shift of Raman band always represents corresponding bond weaken between the vibrating atoms, in turn, the blue shift displays to corresponding bond strengthen^26^. In mitochondria, EtOH weakens not only the -CH=O of TCA but also -CH_3_ and -OH of EtOH itself. A detailed description of the dynamic three dimensional molecular behavior of the interaction between EtOH and TCA needs to be further described by theoretical simulation in the future.

According to the research findings of Mochly-Rosen^17^, mitochondrial aldehyde hydrogenase-2 (ALDH2) must be involved in the process of Et-OH interacting with TCA-CH=O. There are two enzymes,*ε*-protein kinase C (*ε*-PKC)^27^ and ALDH2^28^ *in vivo*. In mitochondria, TCA is induced by hydrogen peroxide. When TCA is generated, EtOH immediately urges *ε*-PKC binding ALDH2, activates ALDH2^29^, then red shifts the Raman band assigned to -CH=O of TCA.

It should be noted that compared with report from Daria Mochly-Rosen^17^, when EtOH (0.5 g/kg, approximately equal to 163 mM) was injected into the abdominal cavity, the ischemia and reperfusion injury of the left anterior descending coronary artery was significantly reduced without causing any side effects *in vivo*. And then combined with the latest research results of this group, resveratrol (0.01 mM) has a significant regulatory effect on the structure of TCA-CH=O for preventing mtDNA damage^30^. Therefore, when the -OH of EtOH changed the structure of TCA-CH=O, its concentration was significantly lower than the safe concentration of ethanol. EtOH for regulating the structure of TCA-CH=O is very safe for human health without any side effects.

In non-hydrogen bonding system, the molecular interaction is very weak. The molecular relaxation time is much longer than the molecular rotation time. The bandwidth of Raman vibration is mainly derived from the fast molecular rotation. But in hydrogen bond system, EtOH as a beneficial solvent, the molecular relaxation time is much shorter than the molecular rotation time due to the strong interaction between molecules. The variations of Raman spectrum mostly come from the fast molecular relaxation^25^.

When Raman band originates from a well isolated vibration, the width of Raman band will be related to only molecular rotation. But in case of a spectrum of a mixture of TCA and EtOH, some individual components have wide and overlapping bands, the broaden width of the band may be simply due to overlapping of bands of EtOH, TCA, and possibly bands from new bonds created between both components as well as molecular rotation of each component. Therefore, the changes of some minor bands should be further explored.

During the interaction of EtOH with TCA, the spectroscopic behaviors of EtOH on TCA were undoubtedly local electron delocalization of TCA-CH=O by -OH in EtOH, especially in mitochondria. But there have been a lot of problems such as the molecular behavior of ALDH2 required to be explored in the future. It is worth noting that the -CH_3_ hydrophobic and steric effects are also very important. On the one hand, the -CH_3_ hydrophobic and steric decreases molecular binding force, declines the activation energy of molecule. On the other hand, the activation energy of -CH_3_ is much weaker than that of -CH=O, and the -CH_3_ rocking motion is faster than that of the -CH=O stretching.

It is known for quite a long time that EtOH is the most safe, common, and important organic solvents, but its other value has not been developed enough. Our results suggested that EtOH should be given high attention, development, and application in other areas. Some compounds containing -OH and -CH_3_ may become effective material for eliminating TCA. The influences of EtOH on TCA-CH=O are pilot contribution to eliminate TCA in order to protect and repair mtDNA by means of the decrease of 8-oxoG.

## Conclusions

The influences of EtOH on the structure of TCA-CH=O were clarified for the first time. It was found that the changes in UV-vis and Raman spectroscopic behaviors of TCA-CH=O can be related to -OH and -CH_3_ of EtOH. Both -OH and -CH_3_ red shifted the maximum UV-vis peak at 301 nm of TCA. Raman stretching band of TCA-CH=O was split by EtOH only related to the -CH_3_ or H in -CH_3_. The -CH_3_ increased TCA-CH=O stretching frequency while the -OH induced it. The more exposed active -OH in polyhydroxy alcohols, the wider FWHM or the less stretching frequency of Raman bands of TCA-CH=O. The ectopic -CH_3_ red shifted UV-vis peak at 301 nm and Raman band of TCA-CH=O. The spectroscopic behavior of -CH_3_ was obviously opposite to that of -OH. In mitochondria, EtOH red shifted Raman stretching band of TCA-CH=O. The C-H bend and rock mode, C-O stretching, and C-C stretching of EtOH itself were red shifted, while the CH_2_ twist and C-C-O bend of EtOH disappeared. The paper unearths the influences of EtOH to trap and transform the structure of TCA-CH=O. This discovery has an important contribution to eliminate TCA in order to protect and repair mtDNA by means of the decrease of 8-oxoG. A detailed description of the dynamic three dimensional molecular behavior of the interaction between EtOH and TCA needs to be further described by theoretical simulation in the future.

## Methods

### Materials

Alkanes were n-hexane, n-heptane, n-octane, n-nonane, and cyclohexane, respectively. Aliphatic alcohols were methanol, EtOH, n-propyl alcohol, n-butyl alcohol, n-pentanol, n-hexyl alcohol, and isopropanol, respectively. Aromatic alcohols were 2-phenylethanol, phenoxyethanol, and benzyl alcohol, respectively. Polyols included glycol, 1, 2- propylene glycol, and glycerol, respectively. Other reagents were potassium chloride, hepes, magnesium chloride, and EDTA. The above reagents were analytical pure without further purification. The purity of all above reagents were not less than 99% and purchased from Beijing Chemical Works (Beijing, China). TCA (97%) was obtained from Shanghai aladdin biochemical technologies limited corporation (Shanghai, China). Fat free bovine serum albumin (BSA) was obtained from Beijing chemical works (Beijing, China). Ultrapure water (18.2 MΩ cm^−1^) was produced using Millipore water purification system.

### Instrument

UV-vis spectrum detection were performed on UV-2550 spectrophotometer (Shimadzu, Japan). The scanning range was from 190 nm to 400 nm with a resolution of 0.1 nm. Raman spectra were detected with Renishaw in Via Raman microscope consisting of a charge-coupled device detector and a confocal digital microscope with a 5×objective, a numerical aperture 0.12, and a 1,800 g mm^−1^ spectrograph gratings. The accumulation time was 10 s. The accumulation number was 5 times. The laser power set on sample was 10 mW. Raman spectra were recorded on an amplified recorder and the 488 nm laser as the excitation source. A spectral range was from 100 cm^−1^ to 1,800 cm^−1^ with a resolution of 1 cm^−1^. The pH value was measured by a PB-10 exact digital pH meter (Sartorius, Germany). Mitochondrial extraction was finished on 3K15 desktop high speed refrigerated centrifuge (Sigma, Germany).

### Mitochondrial preparation

Mitochondria come from rat myocardium. Wistar rats, male, clean grade, were obtained from the laboratory animal center of college of life sciences in Jilin university. Rats weighing 220 ± 2 g were selected. Before animals were euthanized, rats were free to drink and feed for 12 hours and then fast 12 hours, respectively. The heart were removed immediately after animal were anaesthetized with diethyl ether, weighed and immersed in ice-cold mitochondrial isolation buffer. No live animals were tested in this study. Myocardial tissue was obtained from rat heart after procedures approved by the Animal Care and Use Committee of College of Life Sciences, Jilin University, China. All experimental procedures strictly conformed to the Animals (Scientific Procedures) Act 1986 and fully complied with the ethical guidelines for the Care and Use of Laboratory Animals. The buffer was kept cold at 4 °C during the experiment. Other procedures of mitochondrial preparation were operated according to the previous method without modification^31–33^. Mitochondrial samples were used within 1 hour.

### Sample preparations and detected conditions

According to the previous work accumulation, the concentration of TCA was 25 mM. The reference solvent was ultrapure water. The observed UV-vis peak of TCA was located at 301 nm. Choosing the TCA as standard, the observed Raman bands were situated at 1688 cm^−1^ and 1641 cm^−1^, respectively. The samples were freshly prepared and kept in dark place. All samples were detected within 1 hour and the temperature was 25 °C.

### Data image processing

Origin 8.0 software (the United States) was used for data image processing, and the spectra were normalized.

### Data availability

No datasets were generated or analyzed during the current study.

## Electronic supplementary material

Supplementary Information
