# Supplementary material for: Influences of ethanol on the structure of toxic trans-crotonaldehyde in mitochondria coming from rat myocardium
Source: Sci Rep. 2017 Aug 30;7:10081. doi: 10.1038/s41598-017-09656-6 (PMC5577290; doi:10.1038/s41598-017-09656-6)
Supplement: Supplementary file 1 — Supplementary Information [file 41598_2017_9656_MOESM1_ESM.doc]

**Influences of ethanol on the structure of toxic trans-crotonaldehyde in mitochondria coming from rat myocardium**

Yanbin Su^1,†,*^, Xiaowei Ma^2,†^, Yanwen Su^1,†^, Tongxing Mu^3^, Yanhe Li^1^, Ning Jiang^1^, Yuyun Su^1^, and Qi Zhang^1^

^1^College of Chemical & Pharmaceutical Engineering, Jilin University of Chemical Technology, Jilin, 132022, China. ^2^Department of Civil Engineering, Tsinghua University, Beijing, 100084, China. ^3^Lunan Pharmaceutical Group Corporation, Linyi, 276005, China.

*Corresponding author: Yanbin Su, E-mail: 1436547150@qq.com or Suyb620038@jlict.edu.cn

†Yanbin Su, Xiaowei Ma & Yanwen Su contributed equally to the manuscript.

**Supplementary Information**

**1. Raman spectral band assignment of TCA**

**
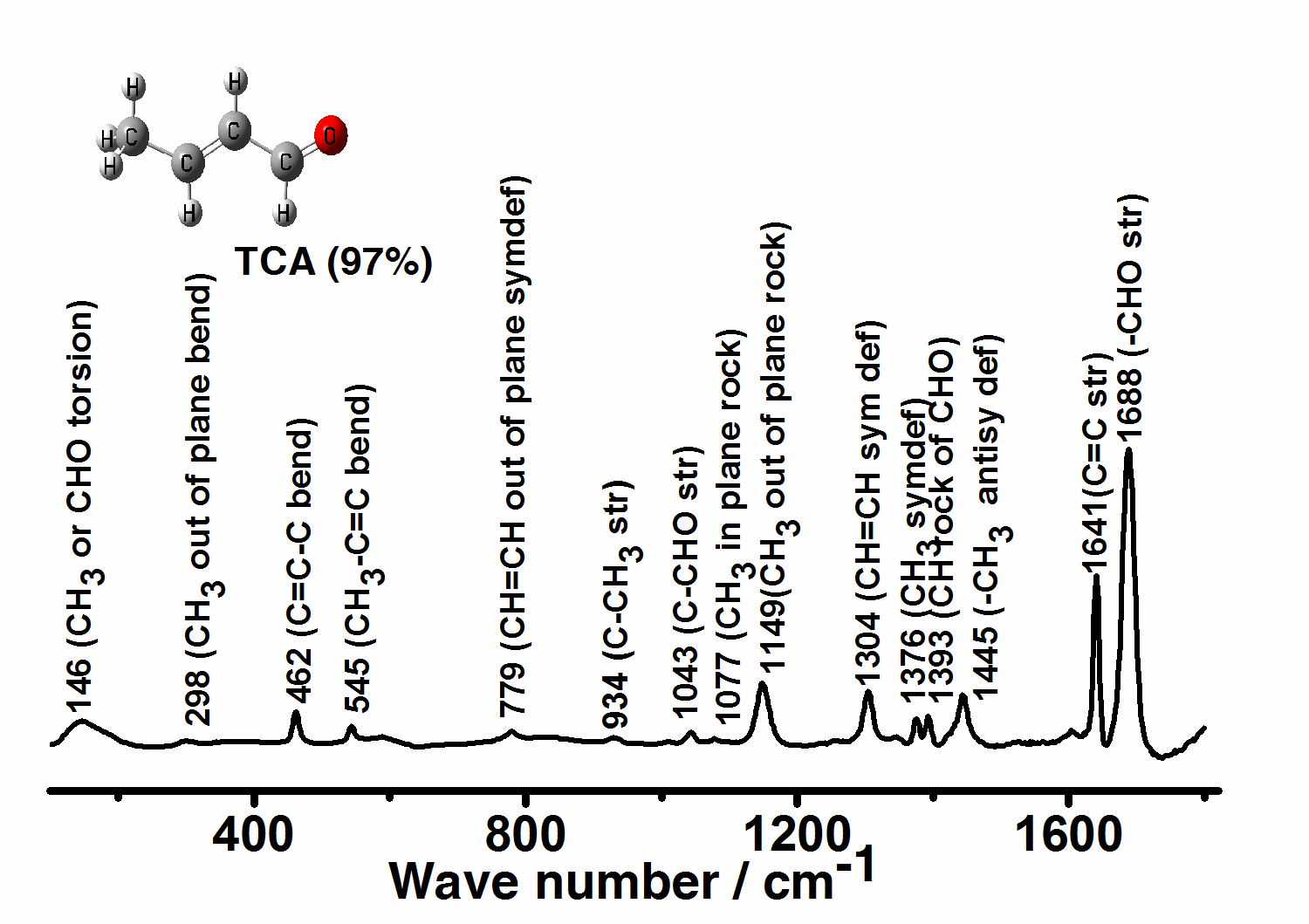
**

**Figure S1.** Raman spectral assignment of TCA

**2. Raman spectra of mitochondria, EtOH, EtOH with mitochondria, TCA, TCA with mitochondria**


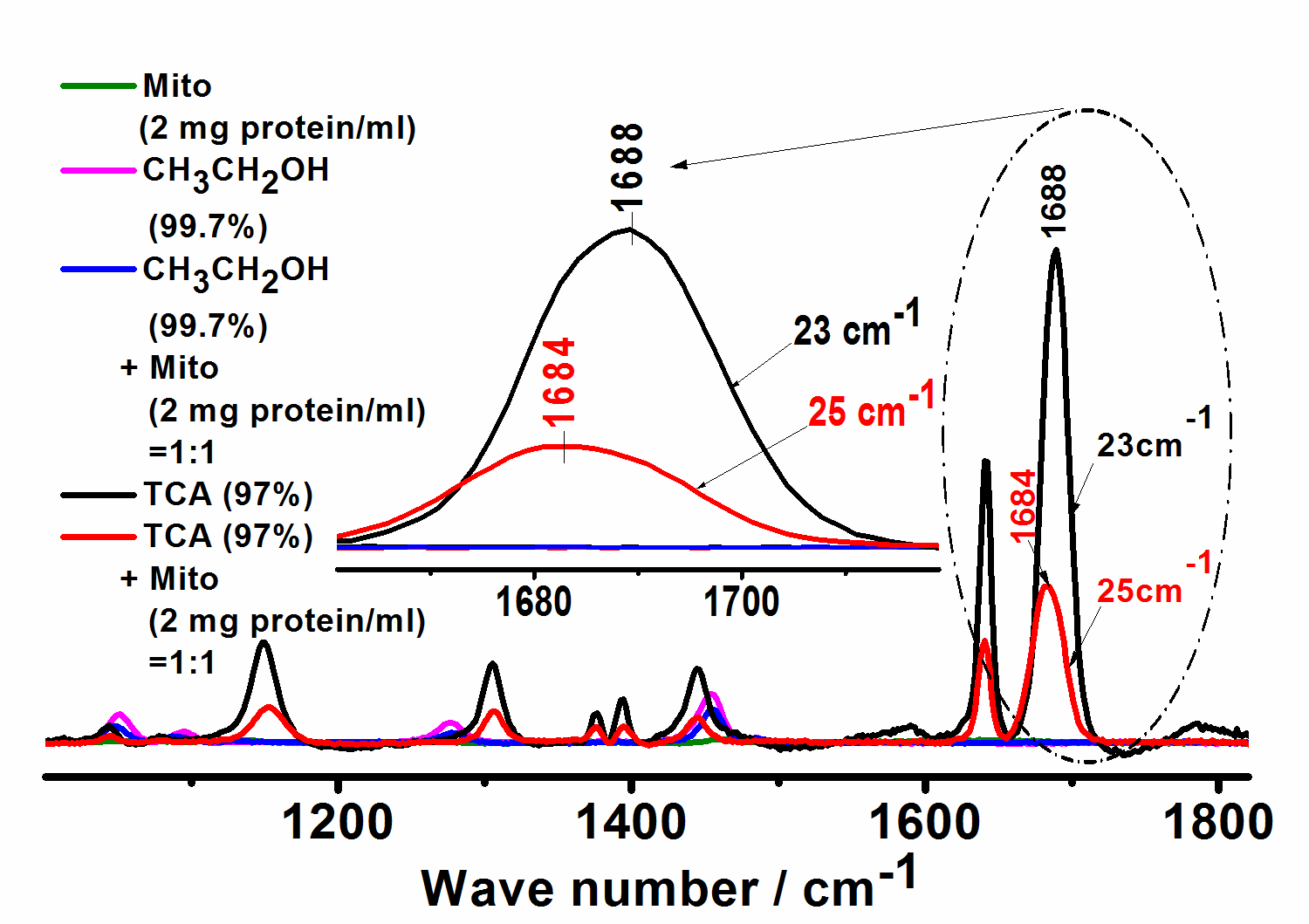


**Note:** EtOH: CH_3_CH_2_OH

**Figure S2.** Raman spectra of mitochondria, EtOH, EtOH with mitochondria, TCA, TCA with mitochondria
